# Effectiveness of the Gold Standard Programmes (GSP) for Smoking Cessation in Pregnant and Non-Pregnant Women

**DOI:** 10.3390/ijerph10083653

**Published:** 2013-08-16

**Authors:** Mette Rasmussen, Berit Lilienthal Heitmann, Hanne Tønnesen

**Affiliations:** 1 WHO-CC Clinical Health Promotion Centre, Bispebjerg and Frederiksberg Hospitals—a part of Copenhagen University Hospital, The Capital Region, Nordre Fasanvej 57, DK-2000 Frederiksberg, Copenhagen, Denmark; E-Mail: hanne.tonnesen@regionh.dk; 2 Institute of Preventive Medicine, Bispebjerg and Frederiksberg Hospitals—a part of Copenhagen University Hospital, The Capital Region, Nordre Fasanvej 57, DK-2000 Frederiksberg, Copenhagen, Denmark; E-Mail: berit.lilienthal.heitmann@regionh.dk; 3 National Institute of Public Health, University of Southern Denmark, Øster Farimagsgade 5A, DK-1353 Copenhagen K, Denmark; 4 Clinical Health Promotion Centre, Department of Health Sciences, Lund University, Skåne University Hospital, Entrance. 108, Malmö SE 205 02, Sweden

**Keywords:** smoking cessation intervention, pregnancy, pregnant smokers, intensive programme, Gold Standard Programme (GSP), effectiveness, abstinence, prospective cohort study, national database, Denmark

## Abstract

*Background*: Smoking is considered the most important preventable risk factor in relation to the development of complications during pregnancy and delivery. The aim of this study was to evaluate the effectiveness of an intensive 6-week gold standard programme (GSP) on pregnant women in real life. *Methods*: This was a prospective cohort study based on data from a national Danish registry on smoking cessation interventions. The study population included 10,682 women of a fertile age. The pregnancy status of the study population was identified using the National Patient Registry. *Results*: The response rate to follow up was 76%. The continuous abstinence rate for both pregnant and non-pregnant smokers was 24–32%. The following prognostic factors for continuous abstinence were identified: programme format (individual/group), older age, heavy smoking, compliance with the programme, health professional recommendation, and being a disadvantaged smoker. *Conclusions*: The GSP seems to be as effective among pregnant smokers as among non-pregnant smoking women. Due to the relatively high effect and clinical significance, the GSP would be an attractive element in smoking cessation intervention among pregnant women.

## 1. Introduction

Worldwide, tobacco smoking is considered to be the second most important risk factor for global disease burden, surpassed only by high blood pressure [1]. In Western Europe, smoking is considered to be the most important risk factor for disease [1]. The severity of smoking is also highly relevant in the case of pregnancy, and in many countries, smoking is considered the most important preventable risk factor in relation to the development of complications during pregnancy and delivery [2]. 

Many studies have established an increased risk of a number of serious complications associated with smoking during pregnancy that can affect both mother and child. These include spontaneous abortion, ectopic pregnancies, perinatal mortality, placental abruption, reduced prenatal development of lung function, preterm birth, low birth weight, conjugate malformations, growth reduction, hospitalisation within the first year of life, and sudden infant death syndrome [2,3,4,5,6,7,8,9,10,11]. Furthermore, a relationship between smoking during pregnancy and the development of behavioural disturbances and lifestyle problems in the child have been shown [2,4,12].

In 2005, the prevalence of women smoking at some point during their pregnancy was 16% on average in Denmark [13], which had decreased from 22% in 1997. From 1997 to 2005 there was a markedly higher smoking prevalence for pregnant women under the age of 25 years [13]. 

Smoking among pregnant women is an issue focused on in the health care sector, and pregnant women are expected to have a higher motivation to quit smoking compared to other groups of smokers. In contrast, other general life conditions may reduce successful quitting, such as being unemployed, having no or very limited education, and being a heavy smoker [14,15], which can all be the case in pregnant women.

Based on this knowledge, it is very important to evaluate how this specific group of smokers can obtain the best help to stop smoking during their pregnancy. This would be of high health importance to the pregnant woman and her child and would be beneficial for the society at large [3].

The aim of this study was to evaluate the effectiveness of intensive smoking cessation interventions (The Gold Standard Programme, GSP) on pregnant women in real life, meaning that the interventions had already been implemented into a normal routine in the smoking cessation clinics. The main hypothesis was that pregnant women undergoing the GSP would be more likely to be smoke-free after 6 months compared to non-pregnant women of the same age. Furthermore, we wanted to identify factors associated with continuous abstinence.

## 2. Methods

### 2.1. Study Design

This study is a prospective cohort study based on data from a national Danish registry (Smoking Cessation Database, SCDB). The SCDB contains data from more than 79,000 smokers who have received face-to-face aid for quitting smoking. Of the face-to-face interventions in Denmark, 80–90% are registered in the SCDB, which is considered to be a representative sample [16]. This project was approved by the Danish Data Protection Agency (2010-41-5463/2000-54-0013) and registered with the Scientific Ethical Committee (H-C-FSP-2010-049). Based on data from the SCDB, we have recently published four papers [16,17,18,19].

### 2.2. Setting

In the study period from 2006–2012, all smokers, including pregnant women, in Denmark had access to more than 230 smoking cessation clinics at different settings, such as hospitals, midwives, municipal clinics, pharmacies, primary care facilities, and other private institutions, that report data to the SCDB. All smokers in Denmark have access to SCIs without referral and free of charge. Less than 5% of this study population chose to participate in a course where they had to pay.

### 2.3. Intervention

Since 2001, the GSP has been the standard smoking cessation intervention (SCI) in Denmark. It comprises 5–6 meetings during six weeks (in a group or as an individual intervention). The programme was based on counselling and a clearly structured manual-based patient education programme taught by specially trained staff [18,19,20,21,22]. The topics of the teaching sessions included ambivalence and motivation, pros and cons of continuous smoking *versus* cessation, setting a quit date, risk situations, withdrawal symptoms and medical support for withdrawal symptoms, relapse prevention and how to handle a completely smoke-free life. Furthermore, the programme contained individual counselling on nicotine replacement therapy (NRT) or other medical support, according to the level of dependence measured by the Fagerström test score [23]. Finally, a hotline was available during daytime hours on working days. The allocation of a single patient to the group or individual programme was at the discretion of the smoking cessation clinics or the instructors. Patients that attended at least 75% of the scheduled meetings were considered to be compliant with the programme as defined by the Steering Committee [17]. 

### 2.4. Participants

From 2006–2012, 27,139 women were registered in the SCDB after giving informed consent. Each woman was registered with a unique 10 digit personal identification number (PIN). The PIN was used to control for double entries, women attending more than 1 intervention, and for identifying pregnant women using data from The National Patient Registry.

Women who were registered in the SCDB at a fertile age (between 15–54 years) and were participating in the GSP in Denmark were included in this study. The fertile age of 15–54 years was determined by finding the youngest and the oldest pregnant smoker registered in the SCDB. The following women were excluded from this study: doublets or entries referring to women attending more than 1 SCI (n = 2,144); women younger than 15 years (n = 38) or older than 54 (n = 8.599); women attending interventions other than the GSP (n = 1,736); women not wanting to be contacted after 6 months (n = 404); and women who were intentionally not followed up with after 6 months (n = 3,536) because the smoking cessation clinic they attended pre-decided not to spend time to follow up on any of their patients.

**Figure 1:**
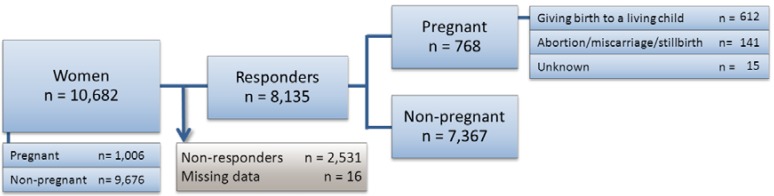
Patient flow. Distribution of women included in the study population according to the follow-up response and pregnancy status. Women 15–54 years of age that were attending the GSP in Denmark between 2006–2012 and attending follow up were included in this study. The non-responders included 2,547 (23.8%) women that were lost to follow up.

Thus, 10,682 women attending a GSP between 2006–2012 in Denmark were included in the study (see Figure 1). Pregnancy (including births, abortions, miscarriages and stillbirths) was identified according to the following ICD-10 codes; FB6601, FB6602, DO00-99, DZ321-DZ39, BKHD4-5, BKKG, BKXG, DN96, DN969, DZ098A, KLCH, KLWW00 and DP95.

### 2.5. Data

Data from SCDB were sought by computer linkage in The National Patient Registry using the 10 digit PINs. Women initiating a SCI from 4 months prior to a registered pregnancy in The National Patient Registry to 12 months after were eligible for inclusion in this study. This time period was chosen to ensure that the SCI was related to a pregnancy. If a woman was registered with more than 1 SCI during the same pregnancy, the most recent one was included in the study. Likewise, if a woman was registered with more than 1 pregnancy, the most recent pregnancy was included.

For all pregnant women with a matching GSP in the SCDB, the following relevant dates were registered; first contact, abortion/miscarriage, birth, stillbirth and/or contact after birth. In total, 9.4% of the responders and 9.3% of the non-responders were pregnant.

### 2.6. Outcomes

The primary outcome was self-reported continuous abstinence for six months, which was defined as not smoking at all from the intended quit date to the six month follow up. The smoking status was obtained by asking whether the patient had been continuously abstinent since the intended quit date reported to the SCDB. If an intended quit date was not reported, the last treatment date was used. The follow up was conducted as a telephone interview after 6 months ± 1 month. After four failed attempts to reach the patient (at least one attempt had to be in the evening), the patient was reported as a non-responder.

Secondary outcomes were abstinence at the end of the programme (self-reported by the patient and observed by the SC counsellor), 14 days point prevalence assessed at the six month follow up by asking the patients if they have been abstinent for the latest 14 days, and patient satisfaction, also assessed at the six month follow up, determined by asking the patients (on a scale from 1–5) how satisfied they were with the course as a whole.

#### 2.6.1. Other Variables

For each patient registered in the SCDB, data were collected on socio-demographic parameters, smoking history, their intervention programme and follow-up information. Age and smoking information were collected as continuous variables. The remaining variables were collected categorically and grouped as shown in Table 1. 

**Table 1:** Characteristics of pregnant and non-pregnant women.

Women in GSP	Pregnant			Non-pregnant	
	Characteristics n (%)			Characteristics n (%)	
**All**	1,006	(100)		9,676	(100)
**Setting**					
**Midwife/Hospital**	438	(43.5)		622	(6.4)
**Other**	568	(56.5)		9,054	(93.6)
**Programme format**					
**Individual**	240	(23.9)		994	(10.3)
**Group**	766	(76.1)		8,682	(89.7)
**Medication for free**					
**No free medication**	511	(50.8)		5,296	(54.7)
**Free for <1 week**	472	(46.9)		4,014	(41.5)
**Free for <5 weeks**	3	(0.3)		75	(0.8)
**Free for ≥5 weeks**	17	(1.7)		279	(2.9)
**Free for unknown period of time**	3	(0.3)		12	(0.1)
**Age (years)**					
**15–24**	240	(23.9)		704	(7.3)
**25–34**	549	(54.6)		1,522	(15.7)
**35–44**	213	(21.2)		3,083	(31.9)
**45–54**	4	(0.4)		4,367	(45.1)
**Smoking**					
**<20 pack-years**	885	(88.0)		4,549	(47.0)
**≥20 pack-years**	121	(12.0)		5,127	(53.0)
**Fagerström 1–4 points**	451	(44.8)		3,658	(37.8)
**Fagerström 5–10 points**	555	(55.2)		6,018	(62.2)
**<20 cigarettes per day**	691	(68.7)		4,739	(49.0)
**≥20 cigarettes per day**	315	(31.3)		4,937	(51.0)
**Heavy smokers ^a^**					
**Yes**	370	(36.8)		6,350	(65.6)
**No**	636	(63.2)		3,326	(34.4)
**Compliance with programme ^b^**					
**Compliant**	476	(47.3)		5,377	(55.6)
**Not compliant**	507	(50.4)		4,121	(42.6)
**Unknown**	23	(2.3)		178	(1.8)
**Living with a smoker**					
**Yes**	523	(52.0)		3,615	(37.4)
**No**	479	(47.6)		5,981	(61.8)
**Unknown**	4	(0.4)		80	(0.8)
**Attempts to quit**					
**No previous attempts**	421	(41.8)		3,616	(37.4)
**Previous attempts**	573	(57.9)		5,896	(60.9)
**Unknown**	12	(1.2)		176	(1.8)
**Professional recommendation**					
**Yes from midwife or medical doctor**	711	(70.7)		3,995	(41.3)
**Yes from others**	42	(4.2)		977	(10.1)
**No**	213	(21.2)		4,265	(44.1)
**Unknown**	40	(4.0)		239	(2.5)
**Education**					
**Low level**	364	(36.2)		2,515	(26.0)
**Medium level**	255	(25.3)		2,552	(26.4)
**High level**	372	(37.0)		4,324	(44.7)
**Unknown**	15	(1.5)		285	(2.9)
**Employment**					
**Employed**	578	(57.5)		6,900	(71.3)
**Unemployed**	277	(27.5)		1,771	(18.3)
**Enrolled in education**	132	(13.1)		671	(6.9)
**Unknown**	19	(1.9)		334	(3.5)
**Disadvantaged smokers ^c^**					
**Yes**	493	(49.0)		3,541	(35.6)
**No**	495	(49.2)		5,757	(59.5)
**Unknown**	18	(1.8)		378	(3.9)

^a^ Heavy smokers: smoking ≥20 pack-year and/or daily consumption of ≥20 cigarettes and/or Fagerström nicotine dependency score of ≥7 point [19,23]; ^b^ Compliance with the programme was defined as having attended at least 75% of the scheduled meetings [17]; ^c^ Disadvantaged smokers: unemployed and receiving unemployment benefits and/or low education (no education except elementary school (≥12 years) or only short work-related courses) [18].

Compliance with the programme was defined as having attended at least 75% of the scheduled meetings [17].

Heavy smokers were defined as patients fulfilling at least one of the following three criteria: smoked at least 20 pack-years, had a daily consumption of 20 cigarettes or more, or had a nicotine dependency of 7 points or more as measured on the Fagerström score [19,23].

Disadvantaged smokers were defined as patients fulfilling at least one of the following two criteria: were unemployed and receiving unemployment benefits, or had a low education level, meaning no education except elementary school (up to 12 years) or only short work-related courses [18].

#### 2.6.2. Sub-Analyses

The following sub-analyses were conducted to establish if there was a difference in the continuous abstinence rates: pregnant women who gave birth to a living child compared to women that had a miscarriage, pregnant women who miscarried compared to non-pregnant women of the same age, older pregnant women (≥25 years) compared to younger pregnant women (<25 years), pregnant disadvantaged smokers compared to non-pregnant disadvantaged smokers of the same age and pregnant heavy smokers compared to non-pregnant heavy smokers of the same age. Finally, a sub-analysis was performed to evaluate the compliance with the programme for non-pregnant women compared to pregnant women.

### 2.7. Statistical Analysis

The data were analysed and reported according to the STROBE recommendations [24]. This included reporting missing data, loss of follow up data and the analyses of the non-responders. Randomised studies usually report the effect of an intervention according to the Russell Standards [25], which assume that non-responders have relapsed. To be able to compare this study to efficacy research, we also reported the continuous abstinence according to these criteria. 

Univariate and multivariate logistic regression analyses were used to test for differences in continuous abstinence. The logistic analyses were adjusted for all of the prognostic factors shown in Table 2. Statistically significant predictors of continuous abstinence were compared by calculating the odds ratio (OR) and the corresponding 95% confidence intervals (CI). Logistic regression analyses were performed by entering all the predictors together. The patients with ‘unknown’ values were excluded from the analyses. These numbers were considered small and acceptable for a real life study. The analyses were repeated for each of the secondary outcomes.

Sub-analyses were performed using the Mantel-Haenszel test for stratified 2×2 tables. A non-responder analysis was performed by comparing the characteristics of responders and non-responders. A two-sided *p* value of <0.05 was regarded as significant. All statistical calculations were performed with StataSE 12.

## 3. Results

The characteristics of the study population showed that non-pregnant and pregnant smokers were different with regard to the following: setting of GSP, programme format (individual/group), age, being a heavy smoker, compliance, living with a smoker, professional recommendation, and being a disadvantaged smoker (education and employment) (see Table 1).

Our study showed that the continuous abstinence rate was not different in pregnant women (24.5–32.0%) compared to non-pregnant women (24.1–31.7%). The following several factors were shown to be important for the abstinence rate: programme format, young age, not being compliant with the programme, being recommended to stop by professionals, being a disadvantaged smoker (without work and/or short-cycle or no education) and being a heavy smoker (see Table 2).

**Table 2:** Primary outcome: ORs with 95% CI for continuous abstinence rates after 6 months for the prognostic factors of the univariate (non-adjusted) and the adjusted multivariable analyses are presented.

Women in GSP		
	Non-adjusted ORs (95% CI)	Adjusted ORs (95% CI)
**Pregnancy**		
**Non-pregnant**	1	
**Pregnant**	1.02 (0.87–1.21)	1.16 (0.95–1.42)
**Setting**		
**Midwife/Hospital**	1	
**Other**	0.94 (0.79–1.12)	0.96 (0.79–1.17)
**Programme format**		
**Individual**	1	
**Group**	0.72 (0.62–0.84)	0.79 (0.67–0.93) *
**Medication for free**		
**No free medication**	1	
**Free for <1 week**	0.87 (0.78–0.96)	0.93 (0.83–1.04)
**Free for <5 weeks**	1.17 (0.69–2.02)	1.07 (0.61–1.88)
**Free for ≥5 weeks**	1.31 (0.98–1.75)	1.22 (0.89–1.66)
**Age (years)**		
**25–54**	1	
**15–24**	0.56 (0.46–0.70)	0.65 (0.51–0.82) *
**Heavy smokers ^a^**		
**Yes**	1	
**No**	1.50 (1.35–1.66)	1.42 (1.27–1.58) *
**Compliance with programme ^b^**		
**Compliant**	1	
**Not compliant**	0.27 (0.24–0.31)	0.29 (0.26–0.32) *
**Living with a smoker**		
**Yes**	1	
**No**	1.16 (1.05–1.29)	1.11 (1.00–1.24)
**Attempts to quit**		
**No previous attempts**	1	
**Previous attempts**	1.13 (1.02–1.25)	1.03 (0.92–1.15)
**Professional recommendation**		
**Yes from midwife or medical doctor**	1	
**Yes from others**	1.24 (1.04–1.47)	1.19 (0.99–1.43)
**No**	1.25 (1.13–1.40)	1.18 (1.06–1.33) *
**Disadvantaged smokers ^c^**		
**Yes**	1	
**No**	1.33 (1.19–1.47)	1.16 (1.04–1.30) *

^a^ Heavy smokers: smoking ≥20 pack-year and/or daily consumption of ≥20 cigarettes and/or Fagerström nicotine dependency score of ≥7 point [19,23]; ^b^ Compliance with the programme was defined as having attended at least 75% of the scheduled meetings [17]; ^c^ Disadvantaged smokers: unemployed and receiving unemployment benefits and/or low education (no education except elementary school (≥12 years) or only short work-related courses) [18]. * Significant. The results were considered significant if the 95% CI did not include the value 1.

### 3.1. Secondary Outcomes

The 14 days point prevalence for being smoke-free after 6 months showed a significant difference between pregnant and non-pregnant women (1.27; 1.05–1.54). No difference was shown between pregnant and non-pregnant women in regard to being smoke-free at the end of the programme or in the satisfaction with the programme (see Table 3).

**Table 3:** Secondary outcomes: Abstinence at the end of the programme, 14 days point prevalence and satisfaction with the programme for pregnant and non-pregnant women (given as the % of all pregnant or non-pregnant patients and responders only). The OR and 95% CI are shown.

Women in GSP	Pregnant				Non-pregnant		
		Effect (%)				Effect (%)	
	Characteristics (n)	All	Responders		Characteristics (n)	All	Responders
**Patients **		1,006	735			9,676	7,252
**Smoke free at end of programme**	419	41.7%	57.0%		4,729	48.9%	65.2%
**OR (95% CI)**	0.98 (0.78–1.21)				1		
**Patients **		1,006	763			9,676	7,319
**Point prevalence (14 days)**	306	30.4%	40.1%		2,801	28.9%	38.3%
**OR (95% CI)**	1.27 (1.05–1.54) *				1		
**Patients **		1,006	742			9,676	7,170
**Satisfied with the programme**	600	59.6%	80.9%		5,853	60.5%	81.6%
**OR (95% CI)**	1.00 (0.79–1.26)				1		

* Significant. The results were considered significant if the CI did not include the value 1.

### 3.2. Sub-Analyses

Furthermore, several sub-analyses were performed. The analysis of disadvantaged pregnant women compared to disadvantage non-pregnant women showed a significantly better continuous abstinence rate for younger pregnant disadvantaged women (2.19; 1.29–3.66). The same effect was not observed for older disadvantaged smokers (0.78; 0.57–1.08). Finally, non-pregnant women were more compliant to the programme compared to pregnant women (1.39; 1.22–1.59).

### 3.3. Non-Responder Analysis

The proportion of non-responders was 23.8%. The characteristics shown in Table 1 were included in a non-responder analysis. The analysis showed that non-responders were significantly more likely to be non-compliant (*p* < 0.001), to be younger (*p* < 0.001), to be disadvantaged (*p* < 0.001) and to have had a SCI in a setting other than a midwife/hospital (*p* < 0.001).

## 4. Discussion

Our study showed that there was a similar effect of the gold standard programme (GSP) on pregnant and non-pregnant women in this large study investigating real life data. Successfully quitting smoking was associated with the individual counselling format, older age, higher compliance, not being a heavy smoker and not having disadvantaged life conditions. When sub-analysing the group of disadvantaged smokers, the programme had a higher effect on the young pregnant women than the young non-pregnant women, while this was not the case in the older group of disadvantaged smokers. Because all smokers in Denmark have access to SCIs without referral and free of charge, we consider the selection bias to be limited.

The abstinence rate in our study was relatively high compared to other studies on pregnant women. During late pregnancy, the rate of smoking cessation has been reported to be 13% (ranging from 7–23%) in a recent review of efficacy and safety of pharmacotherapy for smoking cessation among pregnant smokers [26]. Studies on follow-up from intervention in real life settings have shown similar results [27]. The results in the current study showed a quit rate of 24.5% to 32.2% in pregnant smokers and 24.1% to 31.8% in non-pregnant smokers. From 1997 to 2005 an average of 13.4% of the pregnant smokers in Denmark stopped smoking [13]. Compared to these numbers, the effect of the GSP is very high and seems to be an effective intervention for pregnant smokers. Other studies on the GSP have shown a similar high effect in real life [16,17,18,19]. 

We had expected to find that pregnant women would be more motivated to stop smoking and, thus, have a better quit rate compared to non-pregnant women, but this was only found in a sub-analysis for young disadvantaged pregnant smokers. However, the women still smoking during pregnancy could be a more complex subgroup of all female smokers with special needs and challenges in relation to quitting. This complexity is supported by our findings with regard to the prognostic factors. On one hand, the pregnant women were characterised by positive prognostic factors, such as being enrolled in an individual programme format and lighter smoking in addition to the expected higher motivation caused by the pregnancy. On the other hand, they were also younger than 25 years old, were more often recommended to stop smoking by a health professional, had disadvantaged life conditions and were less often compliant with the programme, all factors related to a lower chance of continuous abstinence. One aspect that explains the similar effect of the GSP among pregnant and non-pregnant women is that these positive and negative factors balanced each other out in the pregnant group. However, other factors not included in the study, such as cultural factors, other lifestyle habits and firmer stress from midwifes and doctors to quit smoking, may be of greater importance. Other aspects that could explain this result are that the expected high motivation for quitting smoking during pregnancy may be overestimated and that the GSP could be relatively robust across socio-economic life conditions, as suggested in a previous study of disadvantaged smokers [18]. 

Several important prognostic factors were identified in the present study, most of which have been previously described to be of similar importance [17,18,19]. In particular, the compliance with the GSP seemed superior to other factors. The successful continuous quit rate doubled for the participants that participated in two visits and doubled again for every subsequent visit, beginning at a few percentages after the first and ending at about thirty percent after participation in all five visits [17]. 

Interestingly, young age seems to be an important prognostic factor with regard to quit rate; women younger than 25 years old were less likely to become smoke free compared to older women. In contrast, other studies of non-pregnant smokers have shown that young smokers are more successful in quitting compared to older smokers [28,29]. 

In spite of the great differences in national smoking prevalence according to age among pregnant smokers [13], our study shows that age *per se* does not have a significant effect on the continuous abstinence rate. From 1997–2005 the smoking prevalence for Danish pregnant women in the age group ≤19 years old, actually increased from 37–43%, while it was relatively stable, approximately 27%, for the 20–24-year-olds [13]. In contrast, the smoking prevalence for pregnant women ≥25 years decreased from approximately 20% to less than 15%.

Nicotine replacement therapy (NRT) is commonly used by the GSP. The recommendation of nicotine replacement therapy for pregnant women has been discussed for many years. Though it might be preferable to stop smoking without nicotine replacement therapy, it is most likely not realistic for the subgroup of pregnant smokers. Recent studies have determined a clear effect of nicotine replacement therapy in sufficient doses for pregnant women in both randomised clinical trials and studies on real life data [26,27]. In Denmark, it is recommended to consider NRT for pregnant women who continue to smoke at least 10 cigarettes daily after three months of pregnancy. The strength of the NRT should be selected according to the usual guidelines for non-pregnant women [30]. This may not be the case in other countries. In this study, 51% of the non-pregnant and 43% of the pregnant smokers used NRT to aid their quitting attempts. The more frequent use of NRT among non-pregnant smokers may be explained by a higher proportion of heavy smokers (see Table 1) as well as the more restrictive guidelines for the recommendation of NRT to pregnant smokers.

This study has limitations as well as strengths. The follow-up rate was relatively high at 76%. The high number of pregnant as well as non-pregnant smokers was a strength of this study. Using continuous abstinence as the outcome measurement of smoking cessation intervention is recommended over point prevalence, as was performed in the present study [31]. The self-reported outcomes were a weakness of this study because patients may overestimate their abstinence rates approximately 3–6% [15,32,33,34]. Therefore, it is possible that pregnant women are more likely to overestimate their success, which could mean that non-pregnant women have a slightly higher quit rate compared to pregnant women. Furthermore, the generalisation of these results should be considered carefully because of different cultural traditions, smoking habits and socio-economic conditions, which should all be taken into account. Further implementation of the GSP in new areas should be followed up to determine if the expected results are obtained.

New research should focus on comparing the GSP with other national, sub-regional or local smoking cessation intervention programs. It would be relevant to evaluate the smoking cessation rates among pregnant women that attended the GSP to that those that did not and to evaluate the smoking cessation rates of their smoking partners in a randomised design. Furthermore, it is necessary to measure the motivation level to quit smoking among pregnant smokers during their pregnancy compared to a non-pregnant control group. It is also necessary to deepen the qualitative interviews to better describe and understand the complexity, special needs and challenges of the pregnant smokers. 

Although smoking has been reduced in many countries, it is still a sizable problem [35] and continues to be a problem among pregnant women. From the literature, we know that between 0.5 and 1% of smokers stop smoking every year in Denmark [16]. A Danish study that included all women that gave birth in 1997–2005, established that the average smoking prevalence among pregnant women decreased from 22% to 16% during that period of time [13], which corresponds to the national decrease in smoking prevalence. The GSP has a high clinical significance because smoking cessation intervention can reduce the occurrence of low birth weight and preterm birth [33]. It is necessary to reach out to all pregnant women who are smokers, and smoking cessation intervention should be given the highest priority by policy makers and health care providers. The relatively high-effective GSP would be an attractive element of a future strategy targeting smoking cessation among all pregnant women. 

## 5. Conclusions

Surprisingly, the Gold Standard programme seems to be as effective among the subgroup of smoking pregnant women as among non-pregnant smoking women. Only in the group of young disadvantaged smokers did the pregnant women show a significantly better continuous abstinence rate compared to the non-pregnant smokers. Successful quitting was associated with the individual intervention format, older age, higher compliance, not being a heavy smoker and not having disadvantaged life conditions. Due to its relatively high effect and clinical significance, the GSP would be an attractive element in future smoking cessation interventions among pregnant women. 
